# Human enteroviruses associated with and without diarrhea in Thailand between 2010 and 2016

**DOI:** 10.1371/journal.pone.0182078

**Published:** 2017-07-27

**Authors:** Jira Chansaenroj, Supansa Tuanthap, Thanundorn Thanusuwannasak, Ausanee Duang-in, Sirapa Klinfueng, Napha Thaneskongtong, Viboonsuk Vutithanachot, Sompong Vongpunsawad, Yong Poovorawan

**Affiliations:** 1 Center of Excellence in Clinical Virology, Faculty of Medicine, Chulalongkorn University, Bangkok, Thailand; 2 Chum Phae Hospital, Chum Phae, Khon Kaen, Thailand; Cincinnati Children's Hospital Medical Center, UNITED STATES

## Abstract

Non-bacterial acute gastroenteritis (AGE) associated with virus infection affects individuals living in developing countries, especially children. To investigate whether shedding of certain human enterovirus (EV) is more frequently detected in the stool of individuals with AGE of unknown etiology than individuals without AGE symptoms, we tested fecal samples collected from 2,692 individuals with diarrhea between January 2010 and December 2016. Samples were tested for rotavirus, norovirus, and EV by reverse-transcription polymerase chain reaction (RT-PCR) and adenovirus by PCR. EV-positive samples were subjected to sequencing and phylogenetic analysis to identify EV species and types. Findings were compared to EV found in 1,310 fecal samples from individuals without AGE who were diagnosed with hand, foot, and mouth disease (HFMD). While the majority of viruses identified in AGE consisted of human rotavirus (22.7%), norovirus (11.4%) and adenovirus (9.3%), we identified EV (6.2%) belonging mainly to species B, C, and rhinovirus. In contrast, >92% of EV found without AGE symptoms belonged to species A. Although AGE symptoms are not often attributed to EV infection, EV was associated with diarrhea of unknown etiology at least in 3.4% of AGE cases. While CV-A6 was most likely to be found in stools of HFMD patients, rhinovirus A and C were the two most common EV species associated with AGE. Elucidating group-specific EV infection in diseases with and without AGE will be useful in assisting identification, clinical management, and the surveillance of EV infection in the community.

## Introduction

Acute gastroenteritis (AGE) caused by viral infection contributes significantly to childhood morbidity and is a leading cause of death in young children [1]. Despite steep declines in mortality associated with viral gastroenteritis in some countries, diarrhea in children resulting in hospitalization still contributes to significant socio-economic burden [2, 3]. Viruses associated with AGE include rotavirus (RV), norovirus (NV) and adenovirus (ADV). RV infection commonly affects children <5 years of age, but has declined in many countries since the introduction of universal RV vaccination program [4]. NV infection has therefore emerged as the leading cause of AGE in this age group [5, 6] with ADV infection not far behind [7, 8]. Nevertheless, the etiology of a significant proportion of gastrointestinal illness remains undetermined especially in developing countries [9].

The association between human enterovirus (EV) infection and AGE is increasingly recognized [10]. Human EV belongs to the *Picornaviridae* family and the *Enterovirus* genus comprising 4 EV species (A to D) and 3 rhinovirus species (A-C) [11]. Collectively, they cause a broad spectrum of acute and chronic diseases especially in infants and young children [12–14]. Coxsackievirus A6 and A16 cause hand-foot-and-mouth disease commonly affecting young children. Symptoms may include mild fever, oral ulcers, and vesicular rash on hands, feet, and mouth [15]. Severe infection by EV such as poliovirus, EV-A71, EV-D68 can result in acute flaccid paralysis, fatal neurological and cardiac complications [16, 17]. EV transmission can be direct via contact with nasal and vesicular discharge or fecal-oral route [18–20], and epidemics can demonstrate a seasonal and cyclical pattern [21, 22]. Identification of the conserved 5’ UTR and/or the viral capsid sequence can differentiate between different EV species and types [23].

Typical EV infection associated with mild skin and oral lesions is the hand, foot, and mouth disease (HFMD). Although HFMD is predominantly caused by members of the EV A species and does not typically result in AGE, some EV are occasionally shed in the stools of patients [24, 25]. Several EV species have been reported to cause gastroenteritis, but the molecular epidemiology of EV linked to diarrhea in children and adults have been limited [15]. To determine whether certain EV species are more often associated with AGE of unknown etiology, we described a multi-year molecular surveillance of EV found in association with AGE compared to EV shed by individuals with HFMD.

## Materials and methods

### Study samples

The study was approved by the Institutional Review Board (IRB) of the Faculty of Medicine, Chulalongkorn University (IRB 491/57 and 286/58). The IRB waived the need for written informed consent because samples were de-identified and anonymous. Permission to use the samples was granted by the Director of King Chulalongkorn Memorial Hospital. Samples collected between January 2010 and December 2016 were categorized on the medical charts as infants (<2 years), pre-school children (2 to <5 years), school-age children (5 to <15 years) and individuals 15 years and older.

The first group of samples consisted of 2,692 stool specimens from individuals ages 3 days to 101 years (mean = 16.2 years; 1,465 males and 1,227 females) with AGE of unknown etiology who sought medical care at hospitals in Khon Kaen province (n = 1,406) and Bangkok (n = 1,286) (S1 Fig). Inclusion criteria were symptoms of watery diarrhea (defined as ≥ 3 episodes within 24 hours) with vomiting and/or fever. These samples were subjected to screening for RV, NV, ADV, and EV.

The second group of samples consisted of 1,310 fecal specimens from patients ages 1 day to 66 years (mean = 3.5 years; 804 males and 506 females) with no AGE symptoms, but had HFMD (defined by blister-like lesions in the buccal cavity, palms, soles, and/or buttocks) [26, 27]. These samples were also obtained from Bangkok (n = 1,060) and Khon Kaen (n = 250) and were tested for EV alone.

### Sample preparation

Samples were suspended in phosphate buffered saline and centrifuged at 4,000 X g for 10 minutes. Viral nucleic acid was extracted from the supernatant using RiboSpin vRD kit (GeneAll, Seoul, Korea) according to the manufacturer’s instructions. The cDNA was synthesized with random hexameric primers using the Improm-II reverse transcription system (Promega, Madison, WI) according to the manufacturer’s instructions.

### Viral detection

#### Rotavirus

RV was detected by RT-PCR to amplify the conserved regions on the VP7 and VP4 genes using SuperScript III One-step RT-PCR system with Platinum Taq (Invitrogen, Carlsbad, CA) as previously described [28]. The VP7 gene was amplified by Beg9 and End9 primers, while Con2 and Con3 primers were used to amplify the VP4 gene.

#### Norovirus

NV was detected using semi-nested PCR to identify the conserved region of the RNA-dependent RNA polymerase and VP1 gene [29]. The PCR was performed using PerfectTaq MasterMix (5 PRIME, Darmstadt, Germany) according to the manufacturer’s instructions. First-round PCR used forward primer JV12y and reverse primer NV2oR, while reverse primer R5591 was used in the second-round.

#### Adenovirus

Semi-nested PCR was used to amplify the ADV fiber gene for initial screening and hexon gene for ADV typing [30] as modified from an earlier study [31]. The expected amplicon size of the hexon gene was 956 bp.

#### Enterovirus

Pan-EV assay using semi-nested RT-PCR to amplify the 5’UTR/VP2 was performed as previously described [32]. Some HFMD samples tested positive for enterovirus have been initially reported [26, 33], but were further characterized in this study. Amplicons from EV-positive samples were agarose gel-purified and sequenced. Nucleotide sequences were analyzed using Chromas Lite (http://www.technelysium.com.au/chromas_lite.html) and Basic Local Alignment Search Tool (BLAST) (http://blast.ncbi.nlm.nih.gov/Blast.cgi). Nucleotide sequences of EV identified from the AGE samples were deposited in the GenBank database under the accession numbers KY079137-KY079263, KR922046, KR054526-KR054554, KY774677-KY774687, and KX349962- KX349964.

### Phylogenetic analysis

Nucleotide sequences of the VP4-VP2 region were aligned and subjected to phylogenetic tree reconstruction using the neighbor-joining method and maximum composite likelihood model implemented in MEGA 5.0 software [34]. Pairwise deletions were utilized for the missing data, and the robustness of the tree was determined by bootstrapping with 1,000 pseudo-replicates. Bootstrap values >70% were considered significant.

### Statistical analysis

Statistical analysis was performed using IBM SPSS V21.0 package (SPSS Institute, Chicago, IL). Chi-square was used to measure differences of EV infection between age groups. The *p* value < 0.05 was considered statistically significant.

## Results

**Fig 1:**
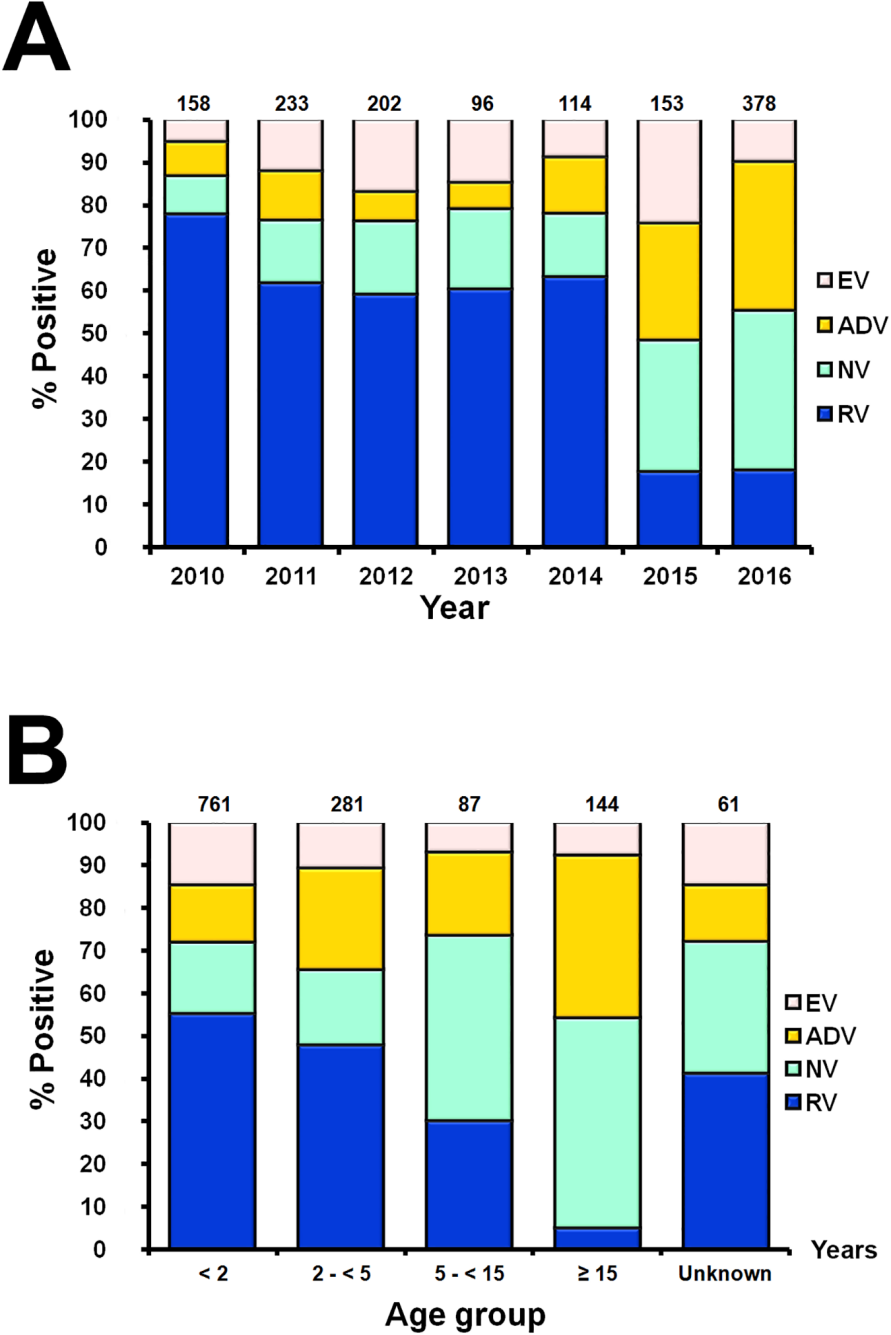
Enteric viruses found in AGE samples from 2010 to 2016. Proportion of EV, ADV, NV, and RV identified by year (A) and by age group (B). The number of virus-positive samples are indicated above the bar graphs. Colors are blue for RV, green for NV, yellow for ADV, and pink, EV.

**Fig 2:**
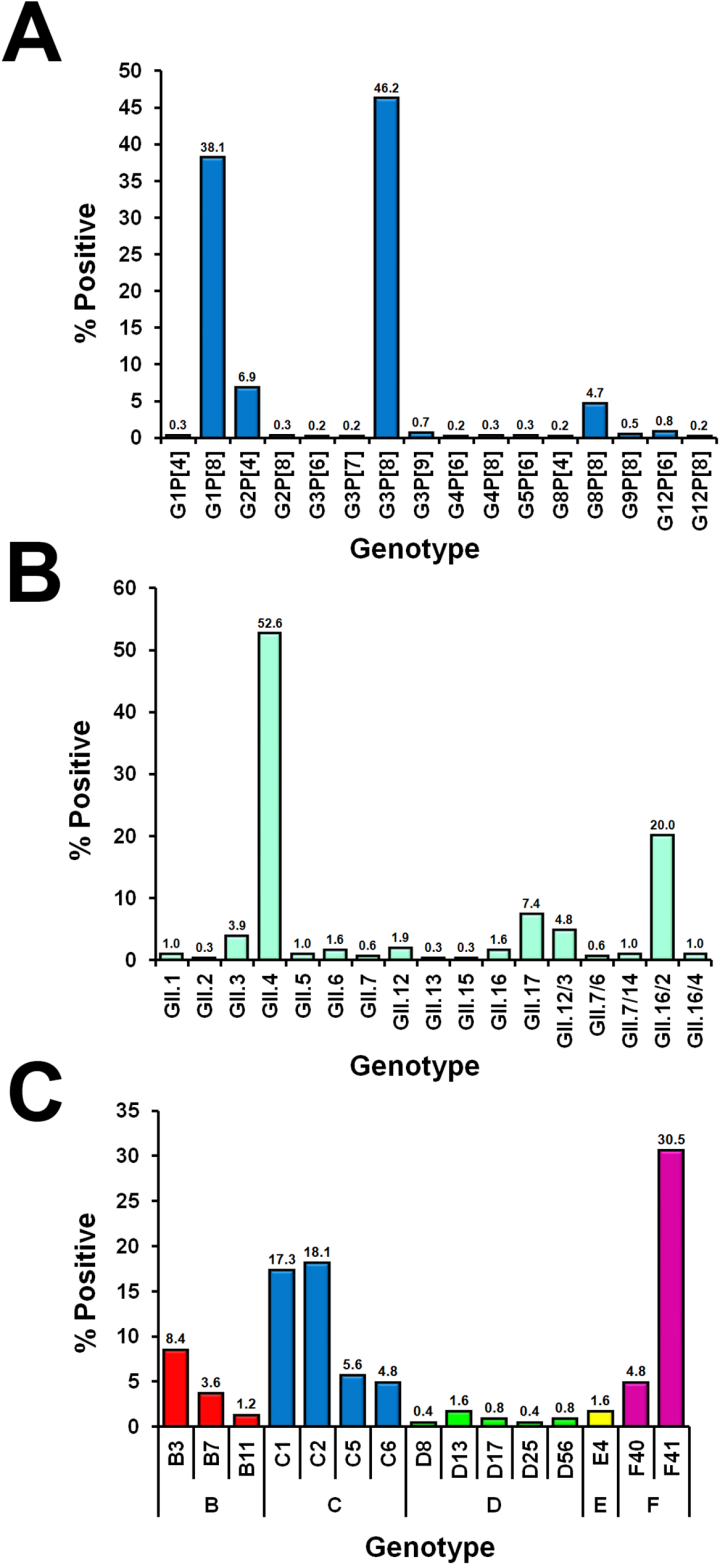
AGE samples tested positive for diverse genotypes of RV, NV, and ADV. The genotype distribution of (A) RV based on the VP7 and VP4 genes, (B) NV based on the RdRp/VP1 region, and (C) ADV based on the hexon gene.

Overall, AGE samples were predominantly from children less than 5 years old (males:females = 1.2:1) (Table 1). Between 2010 and 2016, samples tested positive for RV (22.7%, 611/2,692), NV (11.4%, 306/2,692) and ADV (9.3%, 249/2,692). RV constituted the major virus found in association with diarrhea between 2010 and 2014 (Fig 1A). The prevalent RV genotypes were G3P[8] (46.2%) and G1P[8] (38.1%) (Fig 2A). RV-positive samples were most often found in young children <5 years of age, while NV was more commonly found in older children and adults (Fig 1B). The majority of the NV genotype identified was GII.4 (52.6%) (Fig 2B). In this study, both NV and ADV were major enteric viruses found between 2015 and 2016. Genotype F41 comprised most of the ADV found in the samples (30.5%), followed by C2 (18.1%) and C1 (17.3%) (Fig 2C).

**Table 1:** Characteristics of the cohorts with (AGE) or without (HFMD) diarrhea in this study.

Characteristic	AGE (N = 2,692)		HFMD (N = 1,310)
	N (%)		N (%)
**Gender**			
	Male	1,465 (54.4)	673 (51.4)
	Female	1,227 (45.6)	507 (38.7)
	N/I	0 (0)	130 (9.9)
**Age (years)**			
	< 2	1,214 (45.1)	432 (33.0)
	2 to < 5	370 (13.7)	468 (35.7)
	5 to < 15	157 (5.8)	150 (11.5)
	≥ 15	772 (28.7)	28 (2.1)
	N/I	179 (6.6)	232 (17.7)
**Total EV-positive (N = 168) in each age group**			
	< 2	112 (66.7)	274 (33.5)
	2 to < 5	30 (17.9)	313 (38.3)
	5 to < 15	6 (3.6)	65 (8.0)
	≥ 15	11 (6.5)	13 (1.6)
	N/I	9 (5.4)	152 (18.6)
	Total	168 (100)	817 (100)

N/I = No information on gender or age.

Interestingly, EV was identified either alone or in the presence of other viruses in 6.2% (168/2,692) of the AGE samples (patient mean age = 4.2 years) (S1 Table). In these EV-positive AGE samples, EV was the only virus detected (54.8%, 92/168). Other samples were co-infected with RV (19.6%, 33/168), NV (11.9%, 20/168), ADV (5.4%, 9/168), or with two other viruses (8.3%, 14/168). Meanwhile, EV was detected in 62.4% (817/1,310) of all samples from HFMD, a disease not typically associated with acute diarrhea. Among EV-positive samples in the AGE and HFMD groups, children <5 years of age comprised 84.6% (142/168) and 71.8% (587/817), respectively (*p* < 0.01).

**Fig 3:**
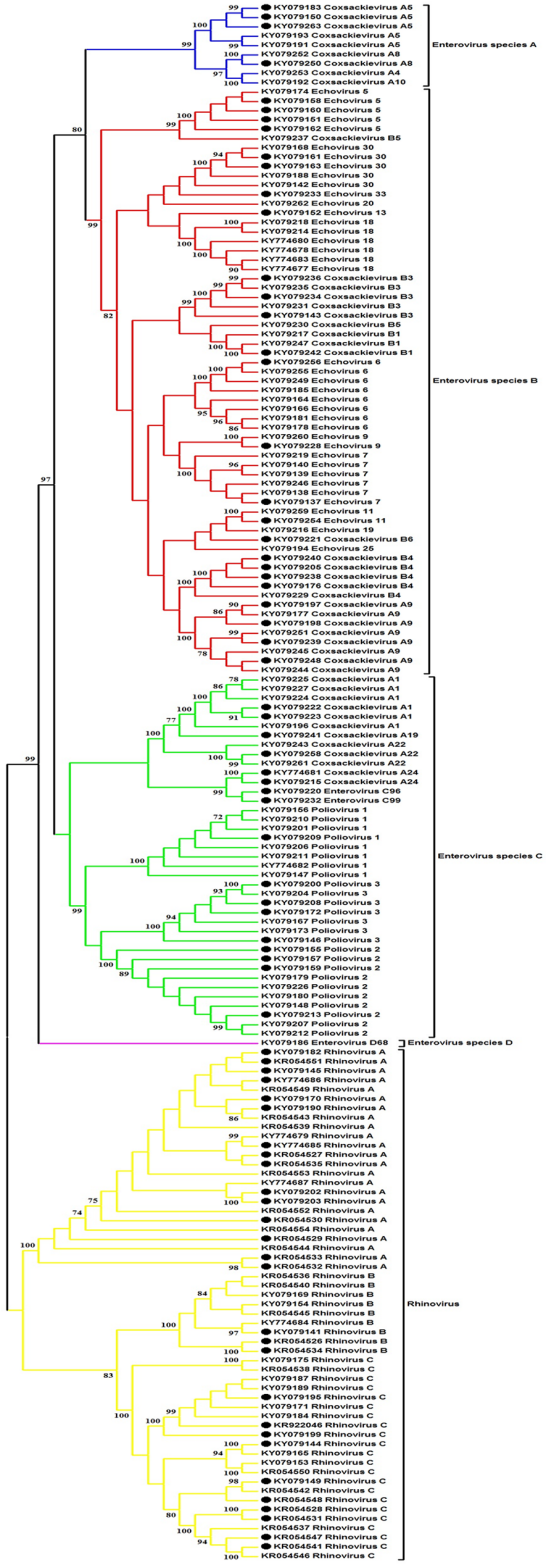
Phylogenetic analysis of the nucleotide sequences of the VP4-VP2 region from EV-positive AGE samples. Phylogenetic tree was constructed using the neighbor-joining method implemented in MEGA (version 5). Bootstrap resampling values >70 are indicated at the nodes. The scale bar indicates the number of substitutions per site. Black dots denote EV obtained from samples with multiple viruses. Blue, EV-A; red, EV-B; green, EV-C; purple, EV-D; yellow, rhinovirus.

**Fig 4:**
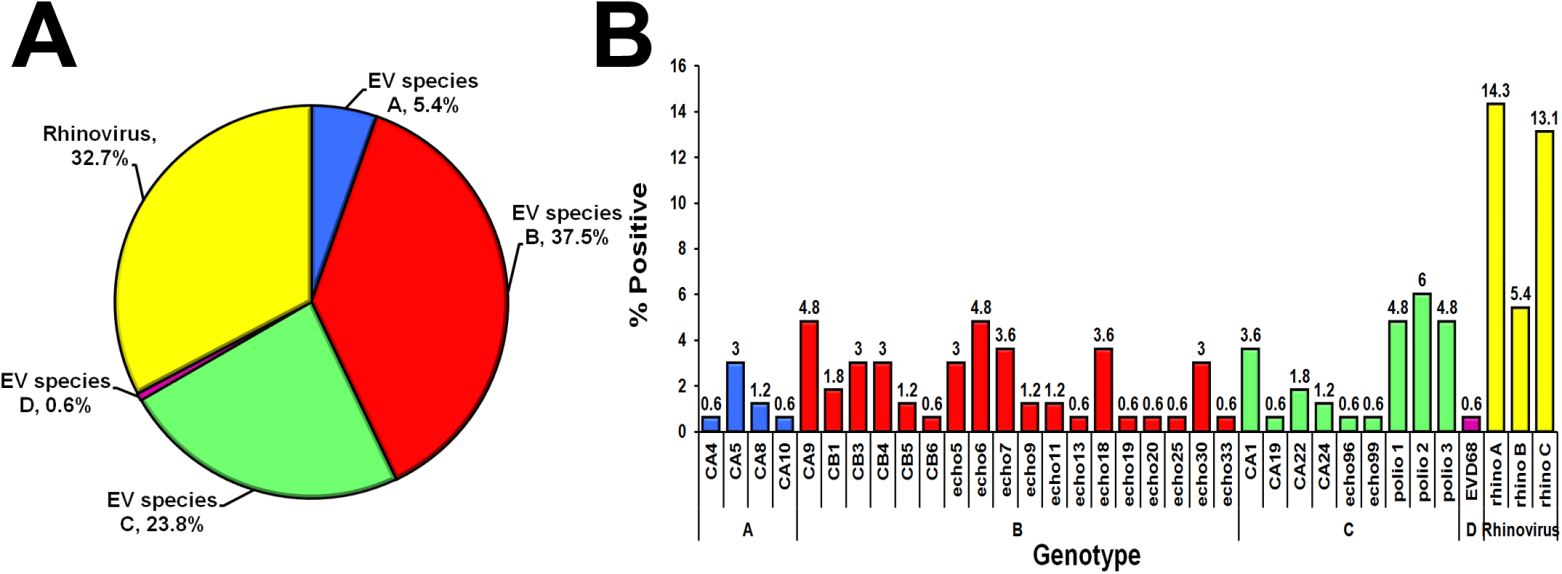
Distribution of EV species and types found in 168 EV-positive AGE samples. (A) Pie chart of EV-A to -D and rhinovirus found in the fecal samples of AGE patients. (B) Genotypes of EV and their percentages (denoted by numbers above the bar graphs). Blue, EV-A; red, EV-B; green, EV-C; purple, EV-D; yellow, rhinovirus.

To further analyze EV in the AGE samples, we performed sequence and phylogenetic analysis. Four human EV species (A-D) and three human rhinovirus species (A-C) were identified (Fig 3). There were 5.4% (9/168) EV-A, 37.5% (63/168) EV-B, 23.8% (40/168) EV-C, and 0.6% (1/168) EV-D (Fig 4A). EV-A comprised genotypes CV-A4, CV-A5, CV-A8, and CV-A10. EV-B species demonstrated the most diversity (18 genotypes), most of which were CV-A9 and echovirus E6 (8/63 for each) (Fig 4B). Of the 9 types of EV-C identified, 26 were Sabin vaccine strains of poliovirus (8 type 1, 10 type 2, and 8 type 3) (S2 Fig). One fecal sample derived from a 3-year-old child tested positive for EV-D68 of clade B2 (S3, S4 and S5 Figs). Finally, rhinovirus was detected in 32.7% (55/168) of the samples (24 species A, 9 species B, and 22 species C), 27 of which were not co-infected with any RV, NV or ADV.

**Fig 5:**
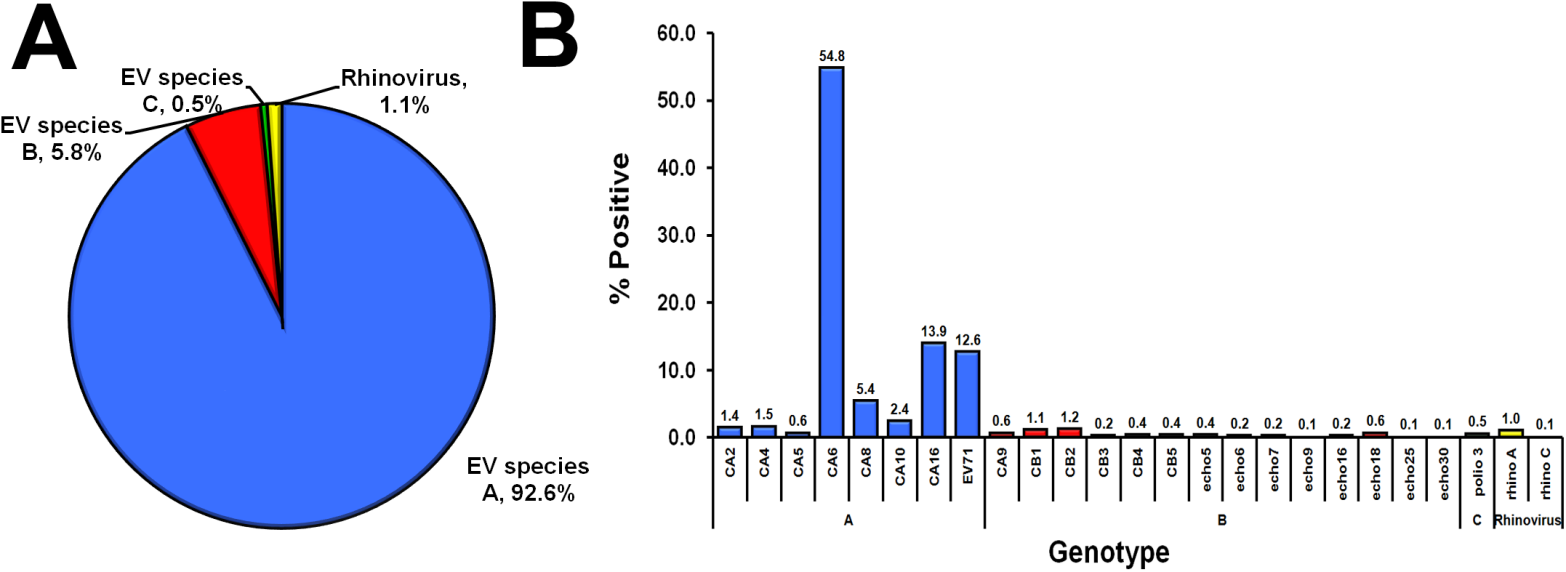
Distribution of EV species and types found in 817 EV-positive HFMD samples. (A) Pie chart of EV-A to -C and rhinovirus found in the fecal samples of HFMD patients. (B) Genotypes of EV and their percentages (denoted by numbers above the bar graphs). Blue, EV-A; red, EV-B; green, EV-C; yellow, rhinovirus.

In contrast, analysis of the EV identified in HFMD samples revealed that an overwhelming majority (92.6%, 757/817) belonged to species A (Fig 5A). Eight EV-A identified were CV-A2, CV-A4, CV-A5, CV-A6, CV-A8, CV-A10, CV-A16, and EV71, most prevalent of which was CV-A6 (54.8%, 448/817) (Fig 5B). Interestingly, CV-A5, CV-A9, echovirus E18 and rhinovirus A were some of the viruses found in multiples samples from both AGE and HFMD cohorts.

## Discussion

Viral gastroenteritis is a significant problem especially among children in under-resourced and developing regions. In addition to affecting the quality of life, AGE imposes a substantial medical and socio-economic burden. Infections caused by RV, NV, and ADV remain significantly underdiagnosed and are responsible for a substantial incidence of diarrhea as was shown in this study and by others [7, 28–30, 35–38]. However, many EVs are also increasingly recognized as being associated with a proportion of persistent diarrhea and are often examined when stools are negative for commonly implicated enteric viruses [14, 19, 35, 39].

In this study, we investigated the molecular epidemiology of viruses typically associated with diarrhea in Thailand. The distribution of the viral etiology of AGE varied by year with RV as the leading cause of diarrhea especially in children below 5 years of age. The gradual increase in RV vaccination in Thailand has contributed to the decline of RV-related AGE and the emergence of NV GII.4 as the leading cause of AGE as was also seen elsewhere [6, 40]. We were particularly interested in the co-detection of EV, especially in samples where RV, NV, or ADV were not detected. Between 2010 and 2016, multiple EV species and genotypes were detected in the AGE samples (6.2%) including EV-B, EV-C, and all 3 species of rhinovirus. In approximately half of these samples (54.8%, 92/168), EV was the only virus present. This represents 3.4% (92/2,692) of all AGE samples, a relatively minor component if compared to RV infection. Comparison of our results to the limited published studies in developing countries with similar tropical climate showed that this rate is lower than the 12.3% of RV-negative, NV-negative, and ADV-negative fecal specimens from children with AGE in northern Ghana [41] and 9.8% in Vietnam [39]. It is also lower than the prevalence of non-polio EV in RV-negative and NV-negative AGE in western India (14.1%) [14]. The lower prevalence of EV-associated AGE observed in this study compared to others may in part be due to the population examined since approximately half of our cohort was from a major urban area of Bangkok. Although we also relied on PCR-based assays, sensitivities and specificities among studies vary. Additionally, viral burdens are expected to be different among different developing countries due to living conditions, diet, and cultural practices.

CV-A6, CV-A16, and EV71 were the three most commonly identified EV associated with HFMD. This finding is consistent with our previous reports [26, 27]. Our study also revealed several interesting observations. For example, the finding of all 3 poliovirus types in 15.5% (26/168) of the AGE samples in this study was not unexpected because children can sometimes experience diarrhea as a result of poliovirus vaccination [42]. One AGE sample from a child with fecal occult blood but no neurological manifestation tested positive for EV-D68. Although EV-D68 causes respiratory infection, detection in stool has been reported [43]. Similarly, all three rhinovirus species were present in the AGE samples despite the absence of other enteric viruses in agreement with a number of other studies [35, 44]. Why nearly half (45.2%) of AGE samples with identifiable enteric virus also had EV is quite puzzling, but the availability of clinical information regarding the severity of diarrhea should enable examination of possible additive effect EV may have on AGE given the diverse genotypes of EV-B and rhinoviruses found in the samples. Finally, the overlapping presence of CV-A5, CV-A9, CV-B1, echovirus E18 and rhinovirus A in both AGE and HFMD samples will require further studies. Of interest are CV-A9, CV-B1, and echovirus E18, which can cause viral meningitis [45, 46]. In addition, CV-A9 and CV-B1 have also been linked to childhood diabetes [47, 48].

There were several limitations in this study. Although it would have been ideal to compare EV found in AGE samples with fecal specimens from age-matched healthy controls who showed no AGE symptoms, a preliminary investigation we performed on 200 fecal specimens from healthy children ≤5 years of age did not show any detectable EV (S1 Fig). We were unable to exclude the possibility that some AGE episodes were caused by bacteria or other less common enteric viruses such as astrovirus, sapovirus, and bocavirus. Although we amplified viral nucleic acid directly from clinical samples, we did not perform confirmation by isolating the viruses using cell culture as was done in some studies. However, we were able to identify one or more viruses in the fecal specimens and verified each with nucleotide sequencing. Future studies involving prospective sequential sampling of stools during healthy and AGE episodes from birth to adulthood may better clarify the burden of EV in diarrheal disease. In summary, the data from this study suggest an association between several EV genotypes and a proportion of AGE cases in Thailand, which underscores the diversity of clinical manifestations afforded by EV.

## Supporting information

S1 Fig Schematic diagram of this study. The presence of EV and other viral pathogens (RV, NV, and ADV) was examined in fecal samples from individuals with and without AGE. EV species identified in the AGE samples were subsequently compared to those identified in HFMD samples collected during the same period.
(TIF)

S2 Fig Phylogenetic analysis of the partial sequence (539 bp) of the 5’UTR/VP2 region of poliovirus vaccine strains found in AGE samples in this study. Black dots denote poliovirus co-identified with multiple viruses in the sample.
(TIF)

S3 Fig Phylogenetic analysis of the partial sequence (539 bp) of the 5’UTR/VP2 region of EV-D68. Black dot indicates the virus identified in this study.
(TIF)

S4 Fig Phylogenetic analysis of the partial sequence (824 bp) of the VP1 region of EV-D68. Black dot indicates the virus identified in this study.
(TIF)

S5 Fig Phylogenetic analysis of the partial sequence (764 bp) of the 3D region of EV-D68. Black dot indicates the virus identified in this study.
(TIF)

S1 Table Identification of EV alone or in the presence of other viruses in the AGE samples. (DOCX)
