# Supplementary figures and images for: Human enteroviruses associated with and without diarrhea in Thailand between 2010 and 2016
Source: PLoS One. 2017 Jul 27;12(7):e0182078. doi: 10.1371/journal.pone.0182078 (PMC5531555; doi:10.1371/journal.pone.0182078)

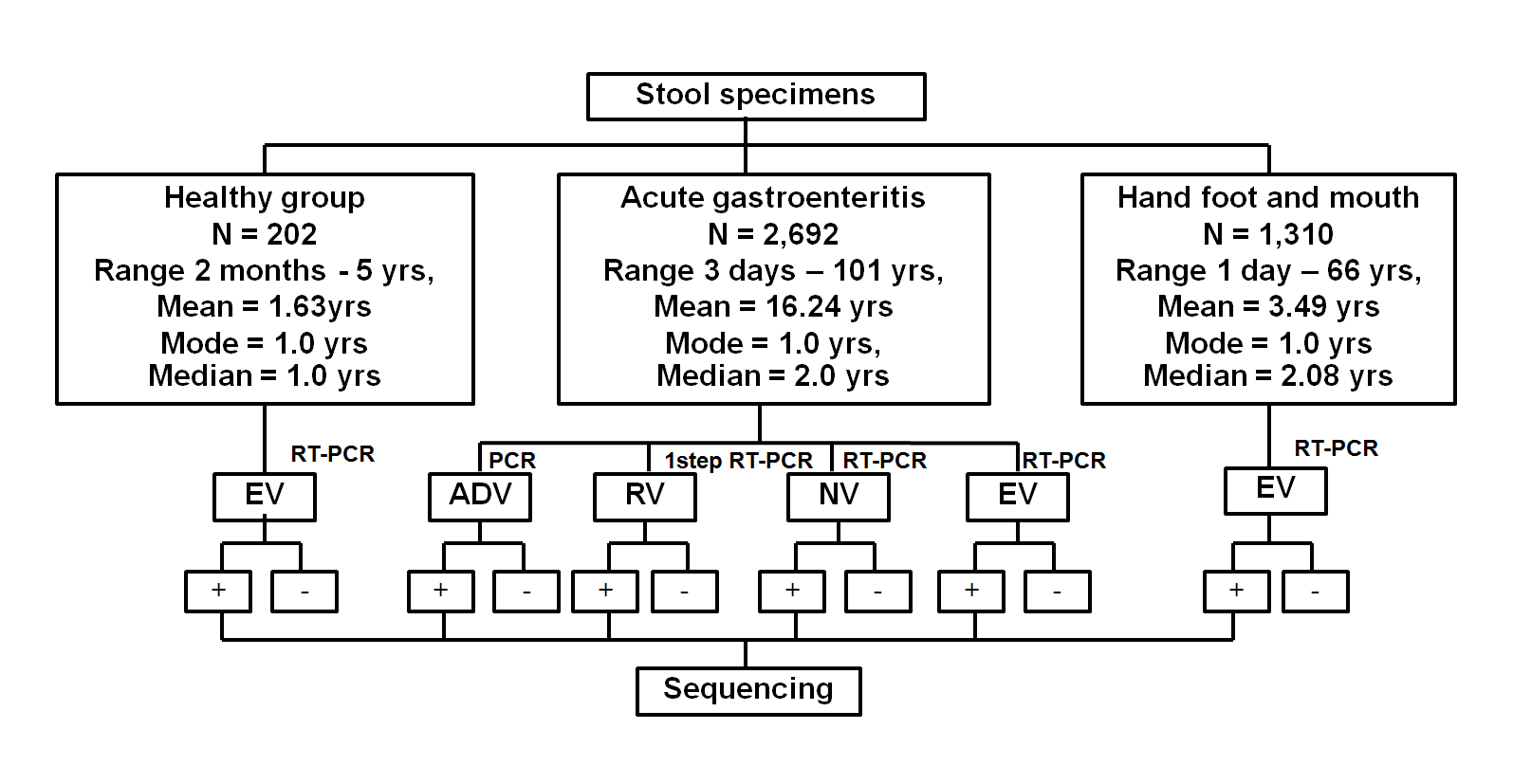

Supplement: S1 Fig — The presence of EV and other viral pathogens (RV, NV, and ADV) was examined in fecal samples from individuals with and without AGE. EV species identified in the AGE samples were subsequently compared to those identified in HFMD samples collected during the same period. (TIF) [file pone.0182078.s001.tif]

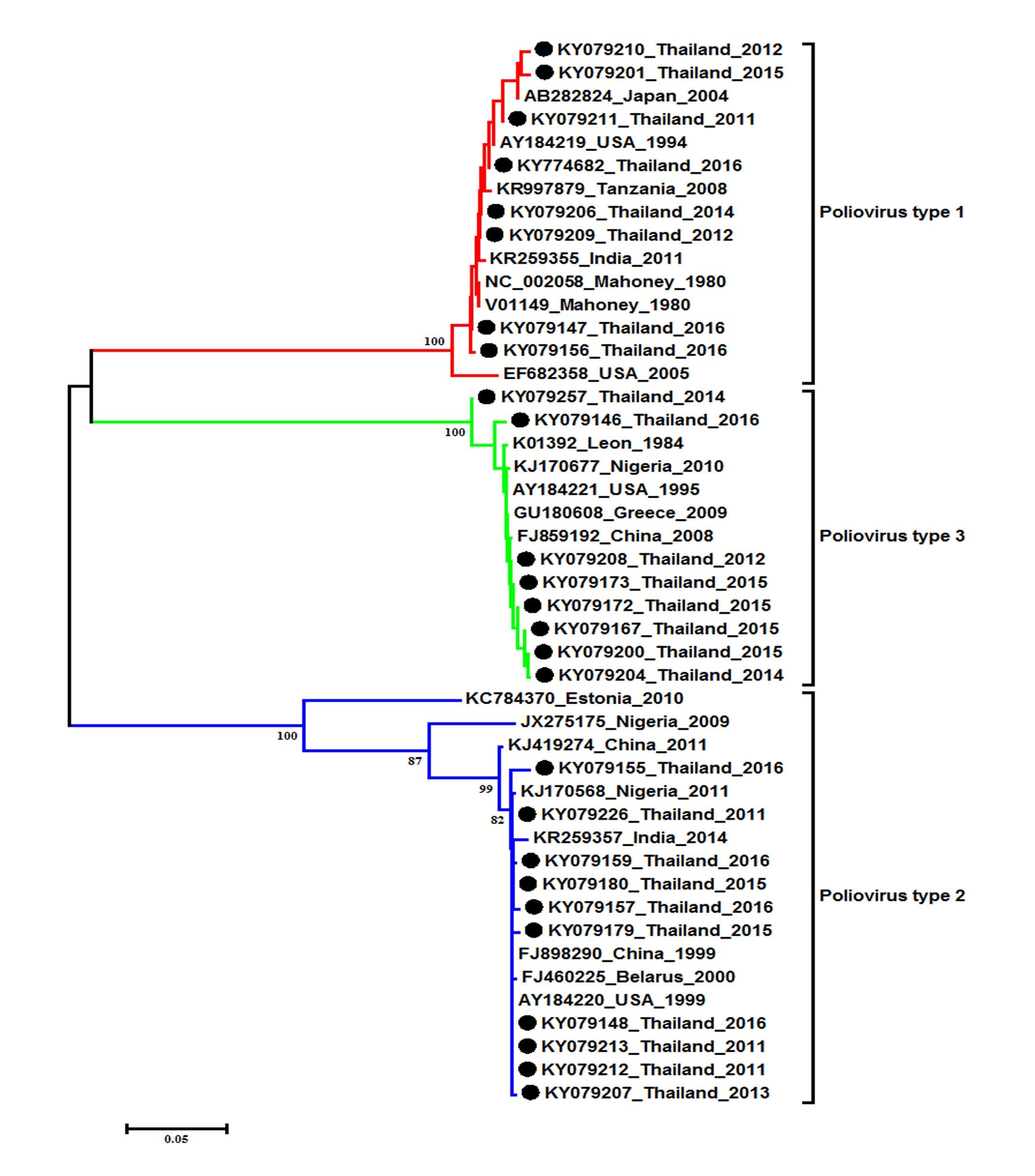

Supplement: S2 Fig — Black dots denote poliovirus co-identified with multiple viruses in the sample. (TIF) [file pone.0182078.s002.tif]

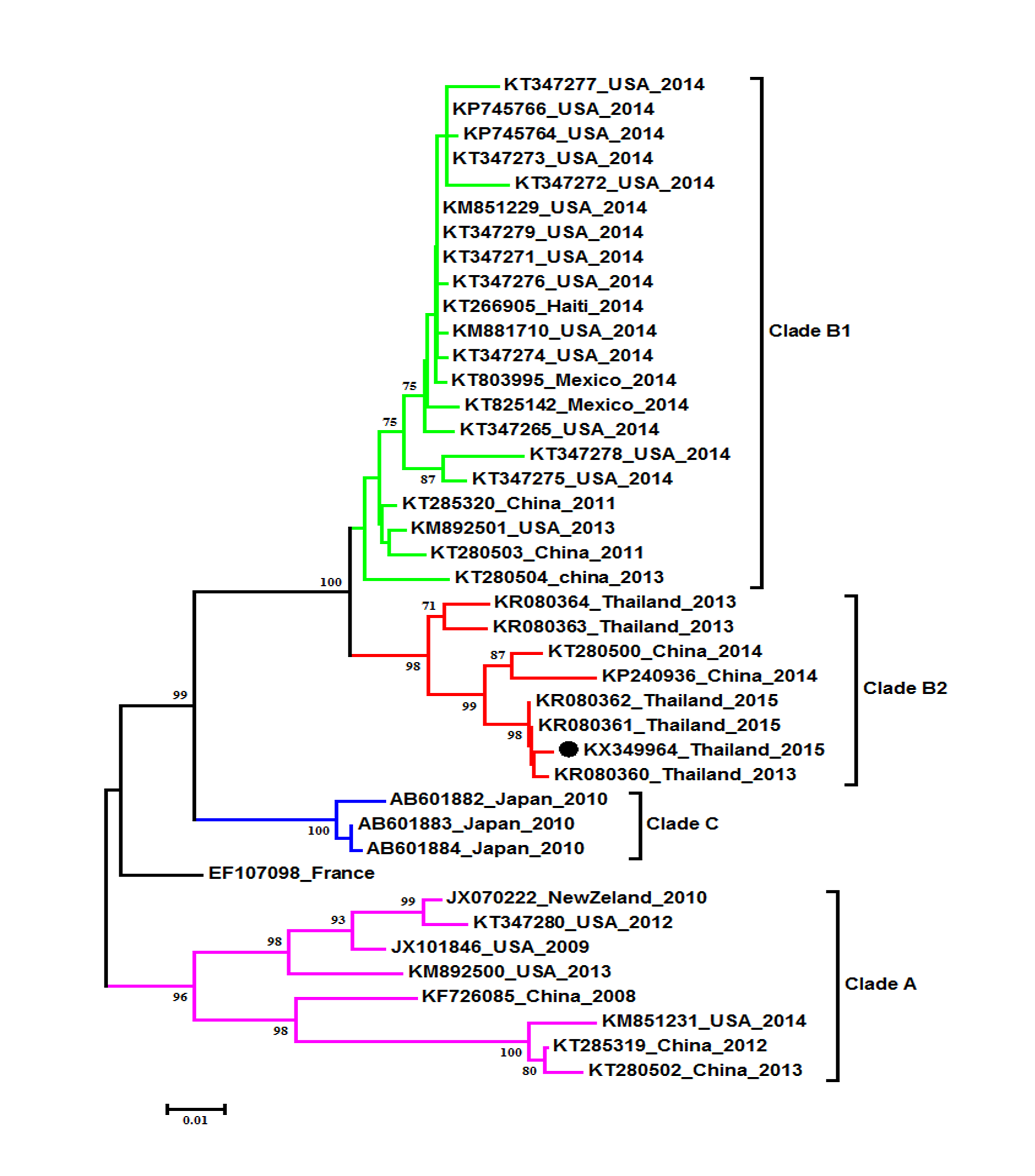

Supplement: S3 Fig — Black dot indicates the virus identified in this study. (TIF) [file pone.0182078.s003.tif]

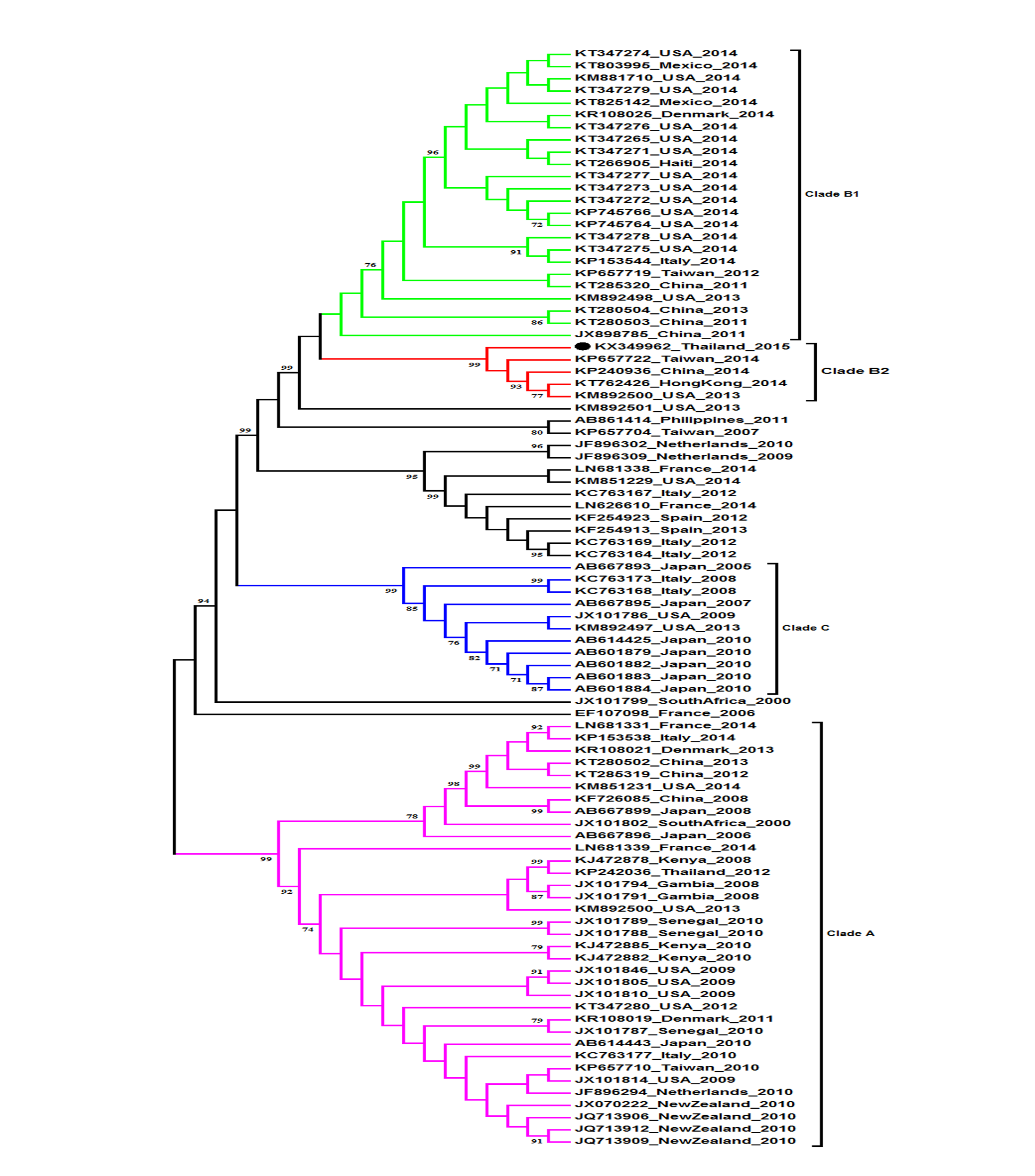

Supplement: S4 Fig — Black dot indicates the virus identified in this study. (TIF) [file pone.0182078.s004.tif]

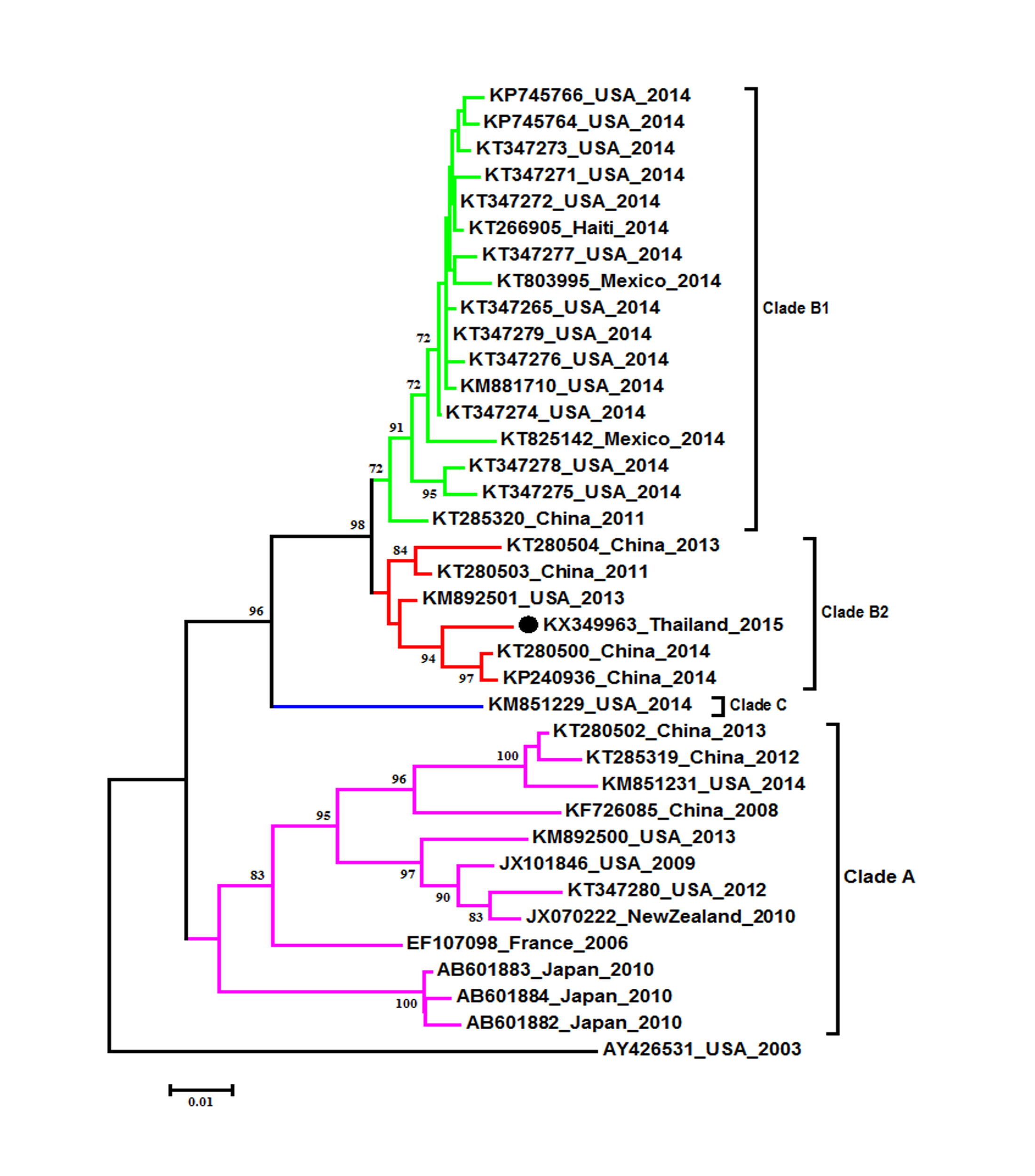

Supplement: S5 Fig — Black dot indicates the virus identified in this study. (TIF) [file pone.0182078.s005.tif]
