# Supplementary material for: Human enteroviruses associated with and without diarrhea in Thailand between 2010 and 2016
Source: PLoS One. 2017 Jul 27;12(7):e0182078. doi: 10.1371/journal.pone.0182078 (PMC5531555; doi:10.1371/journal.pone.0182078)
Supplement: S1 Table — (DOCX) [file pone.0182078.s006.docx]

**S1 Table. Identification of EV alone or in the presence of other viruses in the AGE samples.**

| **Virus, N (%)** | | | | | | | | |
| --- | --- | --- | --- | --- | --- | --- | --- | --- |
|  | **-** | **RV** | **NV** | **ADV** | **RV/NV** | **RV/ADV** | **NV/ADV** | **Total** |
| **EV-positive** | |  |  |  |  |  |  |  |
| EV-A | 5 | 0 | 2 | 2 | 0 | 0 | 0 | 9 (5.4) |
| EV-B | 38 | 12 | 6 | 3 | 1 | 3 | 0 | 63 (37.5) |
| EV-C | 22 | 5 | 6 | 1 | 1 | 4 | 1 | 40 (23.8) |
| EV-D | 0 | 0 | 1 | 0 | 0 | 0 | 0 | 1 (0.6) |
| HRV-A | 10 | 8 | 3 | 2 | 1 | 0 | 0 | 24 (14.3) |
| HRV-B | 6 | 2 | 1 | 0 | 0 | 0 | 0 | 9 (5.4) |
| HRV-C | 11 | 6 | 1 | 1 | 0 | 3 | 0 | 22 (13.1) |
| **Total** | 92 (54.8) | 33 (19.6) | 20 (11.9) | 9 (5.4) | 3 (1.8) | 10 (6.0) | 1 (0.6) | 168 (100) |

EV, enterovirus; RV, rotavirus; NV, norovirus, ADV, adenovirus, HRV, human rhinovirus.
